# Health Literacy of Osteoporosis Risks among Caregivers Serving in Disability Care Facilities

**DOI:** 10.3390/ijerph17134903

**Published:** 2020-07-07

**Authors:** Lan-Ping Lin, Wei-Ju Lai, Shang-Wei Hsu, Jin-Ding Lin

**Affiliations:** 1 Department of Senior Citizen Care and Welfare, Ching Kuo Institute of Management and Health, Keelung 203, Taiwan; lanping518@gmail.com; 2 School of Public Health, National Defense Medical Center, Taipei 114, Taiwan; weiju.lai1990@gmail.com; 3 Department of Healthcare Administration, Asia University, Taichung 413, Taiwan; shangweihsu@gmail.com; 4 Institute of Long-Term Care, Mackay Medical College, New Taipei City 235, Taiwan

**Keywords:** osteoporosis, bone health, health literacy, caregiver, disability institution

## Abstract

Osteoporosis is a global public health issue and its consequent effects are a growing concern worldwide. Caregivers generally experience occupational physical ailments and they have less of a tendency to engage in preventive health behaviors, leading them to be in a higher risk group for osteoporosis. This study aims to present a general profile of health literacy related to osteoporosis risks and identify its associated factors among disability institutional caregivers. A cross-sectional study with a structured questionnaire was used to collect information on 465 caregivers from seven disability care institutions regarding their awareness of the health literacy related to osteoporosis risks. The results indicate that the average literacy score related to osteoporosis risks among the respondents was 60 points (full score is 80 points), with 50–59 being the most common range (51.9%), followed by 60–69 points (43.5%), and 4.4% of cases had more than 70 points. A multivariate logistic regression model revealed that respondents’ age (40–49 vs. 18–29; odds ratio (OR) = 2.53, 95% confidence interval (CI) = 1.31–4.87), education level (senior high vs. primary and junior high, OR = 2.00, 95% CI = 1.03–3.89; college and above vs. primary and junior high, OR = 3.66, 95% CI = 1.84–7.31), experience in undergoing a bone density test (OR = 1.94, 95% CI = 1.28–2.93), and poor physical fitness status (OR = 0.64, 95% CI = 0.43–0.95) were the significant predictors of the osteoporosis health literacy level. The osteoporosis health literacy of institutional caregivers is moderate, and there are many items that are worthy of attention in future health promotion programs. This study highlights risk factors related to a lower level of osteoporosis healthy literacy such as older age, less education, no experience of bone density test, and poor physical fitness that highlight the need to raise further awareness in order to improve caregivers’ bone health.

## 1. Introduction

Osteoporosis and its associated morbidity are growing concerns and are estimated to affect 200 million women worldwide [1]. Osteoporosis is characterized by low bone mass and micro-architectural deterioration of bone tissue, leading to bone fragility and a risk of fracture, disability, and even death [2,3]. Osteoporosis has been highly prevalent but often underdiagnosed and undertreated, to the extent that it has become a silent disease [4]. Enforcing routine health screenings in primary healthcare settings could be an effective strategy to increase osteoporosis awareness and medication use [5]. Providing clear recommendations and encouraging better awareness among general practitioners (GPs) and the general population could improve osteoporosis prevention and treatment. However, most GPs tend to underestimate the salience of osteoporosis [6]. Therefore, reminding health care providers to ensure patients understand osteoporosis risks and encouraging them to participate in preventative behaviors to modify the risk factors is necessary [7].

The effective management of osteoporosis focuses first on reducing modifiable risk factors, such as adopting a balanced diet, adequate calcium and vitamin D intake, adequate exercise, smoking cessation, avoidance of excessive alcohol intake, and fall prevention [8]. Simple educational interventions are valid ways of increasing awareness of osteoporosis among patients with osteoporosis [9], which includes effective education programs related to osteoporosis and enforcing regular exercise activities for improving self-efficacy in osteoporosis prevention [10].

A previous study showed that patients with inadequate health literacy are associated with non-compliance with osteoporosis treatment after sustaining a fracture [11]. As Adami et al. [12] suggested for women at high risk of future fractures, ensuring women’s awareness of their diagnosis and concerns about osteoporosis are critical components in influencing the stage of behavioral transitions in osteoporosis treatment. Thus, it is vital to understand people’s osteoporosis prevention behaviors, such as adequate calcium intake and regular exercise, to maintain healthy bones throughout life [13]. 

The current evidence does not reveal a consistent association between low health literacy and poorer functional outcomes in patients with osteoporosis [14]. However, there is evidence of a care gap between the occurrence of a fragility fracture and the diagnosis and treatment of osteoporosis. People who experience fragility fractures are not likely to receive osteoporosis management for future fracture prevention [15]. An Australian study demonstrated an association between low health literacy and low social economic status, lower levels of education, older age, and anthropometric and lifestyle risk factors for osteoporosis in women [16]. Hill et al. [17] also indicated a substantial burden of low health literacy amongst people with musculoskeletal disease and suggested to enforce the impact of public health education.

Most adults have heard of osteoporosis; however, the majority are not able to accurately describe this chronic condition [18]. Understanding people’s comprehension of osteoporosis might help them to adopt preventive behavior and decrease the burden of disease [19]. Studies on osteoporosis risk perception among caregivers in Taiwan are still limited, particularly in those caregivers who provide assistance and support to the elderly and adults with disabilities. References have shown that about one in ten caregivers report that caregiving has caused their general health to worsen [20], increased rates of physical ailments [21], increased tendency to stress and psychological burdens [22], and serious illness [23]; they also have high levels of obesity and bodily pain [24]. Furthermore, caregivers tend less to adopt preventive health behaviors [25]. These burdens and health risks lead to higher risks of osteoporosis. The study hypothesis will assume that the caregivers’ demographic characteristics, healthy lifestyles, and work patterns are significantly related to their health literacy of osteoporosis risks. Therefore, this study aims to present a general profile of health literacy related to osteoporosis risks and to identify its associated factors among disability institutional caregivers.

## 2. Methods

This study was designed as a cross-sectional study, using a structured questionnaire, to collect institutional caregivers’ awareness of the health literacy of osteoporosis risk. The study population was based on the National Registry of Disability Welfare Services. There are currently 271 disability institutions in Taiwan with 9449 staff members [26]. Due to practical restrictions, after excluding 49 caregivers from the Fujian Disability Welfare Institution (remote island), the population number of caregivers is 9400, and the staff of the disability agency include administrative staff, social workers, nursing staff, education guards, life attendants, trainers, and other personnel. We used Raosoft Inc. [27] statistical webpage to estimate the effective sample size; with a 95% confidence interval and 5% sampling error, the estimated effective sample size is 370 minimally. With regard to the data collection process, firstly we contacted by phone to ask about the willingness of the study setting to participate in the study. Secondly, as the setting agreed to participate in the study, we discussed and determined the number of caregivers to be distributed, and then mailed questionnaires to the responsible contact persons to collect the questionnaires. Finally, this study recruited seven disability institutions and a total of 455 caregivers’ data in the analysis.

For the research ethical considerations, firstly this study received the disability settings agreed to participate in the study after they reviewed the research proposal. The first page of the questionnaire introduced the study’s purpose and right protections to the participant and then the participants signed the informed consent form. This study was anonymous, and the information strictly confidential. In the process of filling out the questionnaire, if the participant felt uncomfortable or do not want to answer, they could withdraw freely from the study at any time.

Firstly, we collected previous literature on osteoporosis research, risk factors of osteoporosis, and related literature on osteoporosis cognition, and then designed a structured questionnaire. The healthy literacy of osteoporosis risks is to understand the knowledge of osteoporosis, the severity of the disease, and the prevention and treatment methods. The structured questionnaire included an informed consent form, demographic, health, and working pattern characteristics of the caregivers, and health literacy of osteoporosis risks (20 questions, Table 1). This study employed an expert’s surface validity (*n* = 5) who reviewed the instrument to determine whether it included all relevant issues and appropriate manners. Face validity can improve the efficacy, readiness, and consistency of a questionnaire. Reliability is the extent to which the questionnaire was stable, dependable, and consistent in the study. The internal consistency reliability test was conducted by IBM SPSS statistical software to determine the overall Cronbach’s alpha coefficient (value is 0.70).

The main data analysis method included descriptive statistics, including the percentage of times to describe the respondents’ characteristics and health literacy of the osteoporosis risk of institutional caregivers. The Chi-squared test was used to explore the correlation test of demographic characteristics, healthy lifestyle, work pattern, and osteoporosis health literacy of caregivers. Then a multiple logistic regression method was employed to explore the possible factors that correlate with the health literacy level of osteoporosis risks in caregivers.

## 3. Results

### 3.1. Characteristics of the Respondents

Table 2 presents data of participants’ characteristics. Of the participants, 16.3% were male and 83.7% were female caregivers who participated in this study. The average age of the participants was 44 years, but most were over 50 years (32.5%), followed by 40–49 years (27.9%), 30–39 years (24.2%), and 18–29 years (15.4%). The average body mass index (BMI) was 24.3 kg/m^2^, and the BMI distribution was mostly in the normal range, with 47.1%, followed by overweight (28.9%), obese (20.5%), and finally underweight (3.5%). In terms of marital status, 30.5% of participants were unmarried and 66.2% were married. Approximately 52.1% of participants had college or above degrees, followed by senior high school (35.2%), and junior high and elementary school degrees (12.7%). In terms of diagnosed diseases of the participants, 32.5% reported that they were diagnosed with a disease(s), and 22.9% needed to take long-term medication. Most of the respondents (55.6%) reported that they were in good shape, and 44.4% reported that they still needed to improve their physical shape in the future.

### 3.2. Health Literacy of Osteoporosis Risks

The caregivers’ total health literacy scale of osteoporosis was 80 points, with the higher scores indicating more knowledge of osteoporosis risks. Table 3 shows that the average score among the caregivers was 60 points, with 50–59 being the most frequent (51.9%), followed by 60–69 points (43.5%), 70 points (4.4%), and only one person (0.2%) with 40–49 points.

### 3.3. Univariate Relation between Respondents’ Characteristics and Osteoporosis Literacy

In this study, there was a cutoff of 60 points for the two groups: high and low level of health literacy of osteoporosis risks. In univariate analyses of the relation between respondents’ characteristics and osteoporosis health literacy (Table 3), two factors—age (*p* = 0.031) and education level (*p* = 0.003)—were significantly correlated with osteoporosis literacy level, and the remaining factors of gender and BMI were not statistically correlated. The analyses in Table 4 show whether there are significant correlations between health literacy level and healthy lifestyle factors, including bone density test (*p* = 0.008) and physical fitness status (*p* = 0.016). The results indicate that those caregivers who have done a bone density test and who maintain good physical fitness have a higher osteoporosis awareness than their counterparts. Other variables such as healthy eating habits (*p*= 0.559), regular exercise (*p* = 0.126), average time of sunshine exposure (*p* = 0.149), and health status (*p* = 0.192) were not significantly correlated with osteoporosis literacy level. In the work status section (Table 5), only job shifts (*p* = 0.047) had a significant correlation with osteoporosis literacy level; no shift caregiver is higher than other shift caregivers, and the front worker (*p* = 0.372), working days (*p* = 0.909), working hours (*p* = 0.088), and work patterns (*p* = 0.563) had no significant correlation with osteoporosis literacy level.

### 3.4. Factors Associated with Health Literacy Level of Osteoporosis Risks

After the statistical tests of univariates, the significant variables were included in the multiple logistic regression analyses of health literacy level of osteoporosis risks (Table 6). The regression model includes demographic characteristics, healthy lifestyles, and work patterns to explore the associated factors of osteoporosis literacy levels; they were as follows: 40–49 years old (odds ratio (OR) = 2.53, 95% confidence interval (CI) = 1.31–4.87), senior high school education level (OR = 2.00, 95% CI = 1.03–3.89), college education and above level (OR = 3.66, 95% CI = 1.84–7.31), those who have undergone a bone density test (OR = 1.94, 95% CI = 1.28–2.93), and those whose physical fitness was poor (OR = 0.64, 95% CI = 0.43–0.95). Like their counterparts, they were still significant predictors of osteoporosis health literacy levels.

## 4. Discussion

Osteoporosis, which is especially prevalent among older postmenopausal women and increases the risk of fractures, particularly of the hip and spine, is associated with high morbidity and mortality in this population [28]. According to the Taiwan Health Promotion Administration (THPA) [29] survey on changes in national nutrition and health, people over 50 years old experience decreased bone density with age and increased osteoporosis, and the prevalent proportion is higher in women than men. Therefore, the THPA has proposed three strategies to “save bones, keep healthy in old age”: (1) keep a balanced diet to maintain more bone health; (2) improve outdoor activities and resistance exercise; (3) understand whether you have osteoporosis “risk factors” to prevent the occurrence. Therefore, how to raise public awareness and understand whether relevant “risk factors” of osteoporosis are the basis for the investigation of osteoporosis health literacy. According to this study, the average score of osteoporosis health literacy among institutional caregivers is fair (60 points, the full score is 80 points). However, it is below the average, and the majority of those with 50–59 points (51.9%) show that there is still room for improvement in the health literacy of caregivers’ osteoporosis. 

Giangregorio et al. [7] stated that people’s perception of risk is influenced by their beliefs in having osteoporosis and their own perceptions of their bone health. For this osteoporosis health literacy survey, caregivers had some misunderstandings or low levels of recognition of osteoporosis risks. Therefore there are many vital issues that deserve special attention in follow-up institutional health promotion programs, such as “osteoporosis is only necessary for women after menopause attention”, “just take calcium tablets regularly can prevent bone loss”, “hormones are not one of the main causes of osteoporosis”, “I think my bones are healthy”, “long-term intense exercise may cause bone loss”, “osteoporosis can cause permanent disability, or even death”, “osteoporosis usually has no symptoms, so people can find it early”, and “lower weight people are more likely to have osteoporosis”. According to a previous study, about 40% of women who reach the age of 50 are expected to suffer from osteoporosis during their lifetime and with consequences such as hip, spinal, or wrist fractures, or death resulting from hip fractures [30]. There is evidence of a care gap between the occurrence of a fragility fracture and the diagnosis and treatment of osteoporosis. The proportion of individuals with a fragility fracture who received an osteoporosis diagnostic test or physician diagnosis ranged from 1.7% to 50% [15]. Therefore, there is still a need to initiate effective public health interventions into osteoporosis prevention to improve people’s bone health. 

In the multivariate logistic regression analysis, results revealed that among the many factors correlated to caregivers’ health literacy level of osteoporosis, “age” and “education level” were two factors significantly correlated with osteoporosis literacy levels. Compared with the previous references, the North American Menopause Society [28] stated that the most common risk factors for osteoporotic fracture are advanced age, low bone mineral density, and previous fracture as an adult. Hage et al. [31] found that women who have never heard of osteoporosis and had a lower level of education had lower knowledge scores. Other studies also found that the knowledge of osteoporosis in postmenopausal women diagnosed with the disease was limited [32,33,34], and level of education was a strong predictor of knowledge [34,35]. In China, Oumer et al. [36] found that the awareness levels for osteoporosis were moderate; lower family income and education level were risk factors for lower awareness. A community-based survey in Saudi Arabia found that women with a low level of education and who had a history of fractures were at high risk of low bone mineral density (BMD) [37]. Therefore, there is a need to improve knowledge of osteoporosis, especially among less educated and minority women, to protect their bone health [38].

The major risk factors for postmenopausal osteoporosis include advanced age, genetics, lifestyle factors (such as low calcium and vitamin D intake and smoking), thinness, and menopause status [28]. This study also found that “has done a bone density test previously” and “good in physical fitness state” are factors that are significantly correlated with the health literacy level of osteoporosis in different lifestyles. The result was similar for Senderovich and Kosmopoulos [39], who found that high-intensity progressive resistance training has been shown to increase vertebral height, femoral neck BMD, and bone reabsorption levels, and to improve bone health. It is suggested that physical fitness and muscle strength are associated with BMD reduction in the lumbar spine, femoral neck, and femur [40].

Other lifestyle issues in individuals with particular osteoporosis risk factors, such as smoking and heavy drinking, are often overlooked for diagnosis and need to be paid attention greater attention in adult populations [41]. Smoking status is suggestive of a role of potential environmental interaction in conferring risk for osteoporosis and the need to focus specifically on its effects [42]. Diet appears to have only a moderate association with osteoporosis [43], but calcium and vitamin D are viewed as safe, natural, and important [44], particularly in older populations [43]. In women, menopause significantly accelerates bone loss and the need to intake adequate nutrition (vitamin D and calcium) and maintain hormone sufficiency during the middle years and beyond [45]. Adequate calcium intake has been shown to reduce bone loss in peri- and postmenopausal women and reduce fractures in postmenopausal women [28]. Other factors, like the sunlight-deprived working environment of institutional caregivers and dietary supplementation of calcium and vitamin D may prove to prevent bone loss and further fractures [30].

This study uses a cross-sectional research method to investigate institutional caregivers’ health literacy and correlated factors. Although this research design has its convenience, there are still many research limitations, including the following: (1) The questionnaire is designed to fill in the signature of the personal consent form. If the participant considers the privacy and sensitivity of the questionnaire content, it may affect the validity of the questionnaire response. (2) The results of this study are the life and work status of the institutional caregiver, which means that the current description of the impact of osteoporosis risk is less able to further explore timing and causality. Despite these limitations, this survey is one of the first in Taiwan to provide a study on health awareness related to osteoporosis in care institutions for people with physical and mental disabilities. The research results provide the organization with an empirical information foundation for the future development of employee health promotion in order to improve their health.

## 5. Conclusions

This study reveals that the osteoporosis health literacy of institutional caregivers is moderate, and there are many items in the healthy literacy scale of osteoporosis risks that are worthy of attention. Based on multivariate logistic regression analyses, we found that many risk factors of a low level of osteoporosis health literacy, such as older age, less education, no experience of bone density examination, and poor physical fitness, raise the need for further awareness to improve individuals’ bone health. Finally, this study highlights that institutional managers should call caregivers to pay attention to osteoporosis risk factors and adopt the recommendations of the THPA [46]. It is advisable to consume balanced and sufficient nutrients, increase sunshine time appropriately, maintain proper weight, not smoke or drink alcohol, and avoid other unhealthy lifestyles at all ages to maintain bone health.

## Figures and Tables

**Table 1:** Health literacy items of osteoporosis risks among the respondents.

1. Osteoporosis only requires special attention for women after menopause.
2. Sunbathing can help vitamin D absorption.
3. Just take calcium tablets regularly to prevent bone loss. *
4. Women are more likely to suffer from osteoporosis than men.
5. Hormones are one of the main causes of osteoporosis.
6. From about 35 years old, people will suffer from bone lose.
7. Young people will not suffer from bone loss. *
8. I think my bones are healthy.
9. I will adjust my diet to prevent bone loss.
10. I will ask the doctor about osteoporosis.
11. Long-term use of steroid drugs can cause bone loss.
12. Correct and moderate exercise can increase bone mass.
13. Long-term intense exercise may cause bone loss. *
14. Osteoporosis patients need to avoid fractures due to falls.
15. Osteoporosis can cause permanent disability and even death.
16. Bad habits, such as smoking and drinking, will increase the chance of suffering from osteoporosis.
17. Osteoporosis usually has no symptoms, so people cannot find it early.
18. Keep bone healthy as much as possible to prevent osteoporosis when young.
19. The best way to treat osteoporosis is to take medicine. *
20. People with lower body weight are more likely to have osteoporosis.

* reverse question.

**Table 2:** Characteristics of the respondents and total score of health literacy.

Variables	*n*	%	Mean ± SD (Range)
Gender			
Male	74	16.3	
Female	381	83.7	
Age			43.5 ± 11.5 (19.6–75.8)
18–29	70	15.4	
30–39	110	24.2	
40–49	127	27.9	
≥50	148	32.5	
BMI			24.3 ± 4.0 (15.8–40.8)
Underweight	16	3.5	
Normal	214	47.1	
Overweight	131	28.9	
Obese	93	20.5	
Marital status			
Unmarried	139	30.5	
Married	301	66.2	
Other	15	3.3	
Education			
Primary and junior high	58	12.7	
Senior high	160	35.2	
College and above	237	52.1	
Diagnosed diseases			
No	307	67.5	
Yes	148	32.5	
Medication			
No	351	77.1	
Yes	104	22.9	
Physical fitness			
Good	253	55.6	
Poor	202	44.4	
Total score of health literacy			
40–49	1	0.2	60.3 ± 5.0 (48–77)
50–59	236	51.9	
60–69	198	43.5	
≥70	20	1.1	

SD: standard deviation; BMI: body mass index: underweight <18.5, normal 18.5–23.9, overweight 24.0–26.9, obese ≥27.0. Low level of health literacy: score 40–59; high level of health literacy: score ≥60.

**Table 3:** Univariate relations of health literacy level and demographic characteristics.

Demographic Characteristics	Low (<60)	High (≥60)	*χ*^2^ Test
	*n* (%)	*n* (%)	*p*-Value
Gender			0.165
Male	44 (59.5)	30 (40.5)	
Female	193 (50.7)	188 (49.3)	
Age			0.032
18–29	39 (55.7)	31 (44.3)	
30–39	63 (57.3)	47 (42.7)	
40–49	52 (40.9)	75 (59.1)	
≥50	83 (56.1)	65 (43.9)	
Education			0.002
Primary/junior high	40 (69.0)	18 (31.0)	
Senior high	90 (56.3)	70 (43.8)	
College and above	107 (45.1)	130 (54.9)	
BMI			0.079
Underweight	4 (25.0)	12 (75.0)	
Normal	108 (50.5)	106 (49.5)	
Overweight	76 (58.0)	55 (42.0)	
Obese	48 (51.6)	45 (48.4)	

**Table 4:** Univariate relations of health literacy level and healthy lifestyle.

Healthy Lifestyle	Low (<60)	High (≥60)	*χ*^2^ Test
	*n* (%)	*n* (%)	*p*-Value
Health status			0.175
Excellent	27 (64.3)	15 (35.7)	
Good	86 (47.0)	97 (53.0)	
Fair	107 (54.6)	89 (45.4)	
Poor/bad	17 (50.0)	17 (50.0)	
Bone density test			0.008
Yes	121 (46.7)	138 (53.3)	
No	116 (59.2)	80 (40.8)	
Physical fitness			0.016
Good	119 (47.0)	134 (53.0)	
Poor	118 (58.4)	84 (41.6)	
Healthy eating			0.343
Very unhealthy/unhealthy	85 (55.2)	69 (44.8)	
Healthy/very healthy	152 (50.5)	149 (49.5)	
Regular exercise			0.126
No	74 (57.8)	54 (42.2)	
Yes	163 (49.8)	164 (50.2)	
Sun exposure daily			0.149
<30 min	76 (47.5)	84 (52.5)	
≥30 min	161 (54.6)	134 (45.4)	

**Table 5:** Univariate relations of health literacy level and working pattern.

Working Conditions	Low (<60)	High (≥60)	*χ*^2^ Test
	*n* (%)	*n* (%)	*p*-Value
Front worker			0.372
Yes	170 (53.5)	148 (46.5)	
No	67 (48.9)	70 (51.1)	
Working days weekly			0.909
≤5	161 (52.3)	147 (47.7)	
>5	76 (51.7)	71 (48.3)	
Working hours daily			0.088
≤8	187 (54.4)	157 (45.6)	
>8	50 (45.0)	61 (55.0)	
Shift work			0.047
Yes	111 (57.5)	82 (42.5)	
No	126 (48.1)	136 (51.9)	
Work pattern			0.498
Static/static mostly	44 (47.3)	49 (52.7)	
Half static and half dynamic	104 (54.7)	86 (45.3)	
Dynamic/dynamic mostly	89 (51.7)	83 (48.3)	

**Table 6:** Multiple logistic regression of health literacy level of osteoporosis risks (*n* = 455).

Variable	OR (95% CI)	*p*-Value
Age (30–39 vs. 18–29)	1.07 (0.57–2.01)	0.835
Age (40–49 vs. 18–29)	2.53 (1.31–4.87)	0.006
Age (≥50 vs. 18–29)	1.49 (0.76–2.95)	0.247
Education (senior high school vs. primary/junior high school)	2.00 (1.03–3.89)	0.041
Education (college and above vs. primary/junior high school)	3.66 (1.84–7.31)	<0.001
Bone density test (yes vs. no)	1.94 (1.28–2.93)	0.002
Physical fitness (poor vs. good)	0.64 (0.43–0.95)	0.028
Shift work (no vs. yes)	1.49 (1.00–2.22)	0.052
